# Knowledge of Impacted Teeth among the Undergraduate Dental Students of a Medical College: A Descriptive Cross-Sectional Study

**DOI:** 10.31729/jnma.6385

**Published:** 2021-07-31

**Authors:** Rajesh Twyana, Pranaya Khanal, Bikash Chaudhary, Alok Sagtani, Sujaya Gupta

**Affiliations:** 1 Department of Oral and Maxillofacial Surgery, Kathmandu Medical College and Teaching Hospital, Duwakot, Bhaktapur, Nepal; 2 Department of Periodontics and Oral Implantology, Kathmandu Medical College and Teaching Hospital, Duwakot, Bhaktapur, Nepal

**Keywords:** *dental students*, *impacted tooth*, *knowledge*

## Abstract

**Introduction:**

Third molar impaction is a common patient complaint in dentistry. Common symptoms are localized pain, swelling, bleeding, and difficulty in mouth opening. Since dental students deal with patients early in their education, for better skill, they should have knowledge of various teeth impactions. Hence, the objective was to find out the knowledge of impacted teeth among undergraduate dental students of a medical college.

**Methods:**

A descriptive cross-sectional study was conducted among bachelor in dental surgery students of a medical college rom November 30, 2020 to April 3, 2021. Data collection was done by convenience sampling after receiving ethical clearance from Institutional Review Committee (Reference 1208202006). A structured questionnaire in Google Forms was sent to 221 participants via Viber. Out of 213 responses received, 144 were analyzed in Excel after discarding for duplication and other errors. Descriptive statistics such as mean, standard deviation, frequency, and percentage were analyzed.

**Results:**

Out of the total of 144 participants, only 81 (56.3%) (47.89-91.4 at 95% Confidence Interval) had known about the term 'impacted teeth' before joining Bachelor in Dental Surgery course. The source of information was mostly the internet 27 (18.8%) followed by dentist 23 (16%). Most students 116 (80.6%) were familiar with third molar impactions, 62 (43.1%) knew about types of impacted teeth/impaction, and 100 (69.4%) were aware of the complications of not removing impacted teeth.

**Conclusions:**

Dental students should be provided with appropriate 'impacted teeth' education supported by practical experience. More detailed information regarding impacted teeth should be included in the curriculum for better understanding.

## INTRODUCTION

Third molars or wisdom teeth generally erupt from 17 to 25 years of age.^1^ Most adults have four wisdom teeth, but can have less or more. Third molars are the most commonly impacted teeth and account for 98% of all impactions.^2^

The most common symptoms with third molar impactions are localised pain, swelling, and bleeding which are due to pericoronitis. Pericoronitis is inflammation of gingival overgrowth, the operculum.^3^ Food particles and bad oral hygiene can predispose to caries and further aggravate the symptoms. Pain due to impaction can radiate to ear, temporomandibular joint, and posterior submandibular region. Furthermore, trismus (limited mouth opening) can occur as complication.

The undergraduate dental students deal with patients early in their curricular clinical education. Thus, for better skill, they should be provided with appropriate impacted teeth education and practical knowledge. Hence, the aim was to assess the knowledge of impacted teeth among undergraduate dental students.

## METHODS

A descriptive cross-sectional study was conducted among the Bachelor of Dental Surgery (BDS) students of Kathmandu Medical College and Teaching Hospital (KMCTH), Kathmandu, Nepal after obtaining ethical clearance from Institutional Review Committee (Ref. 1208202006). The BDS students of first year to fifth year were included in the study while those BDS students undergoing internship and undergraduate students of other KMCTH programs were excluded. The data collection was done from November 30, 2020 to April 3, 2021 and participants were recruited by convenience sampling technique. The sample size (n) of 140.21≈141 was calculated using following formula:

n_o_ = Z^2^ × p × q / e^2^

  = (1.96)^2^ × (0.53) × (0.47) / (0.05)

  = 383

Where,

n_o_ = sample size
Z = 1.96 at 95% Confidence Interval
p = prevalence, 53% (percentage of first year BDS students who were familiar with third molar impaction)^4^
q = 1-p
e = margin of error, 5%

The sample size is adjusted by using the formula,

n = n_o_ / 1+[(n_o_ - 1)/N]

  = 383 / 1+[(383-1)/221]

  = 140.21

Where,

n = adjusted sample size
N = total number of BDS students at KMCTH, 221

A structured self-administered questionnaire from previous study^4^ consisting of 11 closed-end questions were framed in two sections. In the first, informed consent was obtained and the participants had to answer three questions for demographic information and in the second part, questions regarding knowledge of impaction and impacted teeth were included. The questionnaire was prepared on “Google Forms” and distributed through social networking application, Viber. “Google Forms” is a tool for conducting surveys, tests, or web input forms. The information is then automatically collected in Google Drive which is connected to a spreadsheet, where results can be tracked and stored on the web without having to know complicated programming and analytical software. The questionnaire was filled by 213 participants but after discarding for duplication and other errors, only 1 44 forms were included for final analysis. Microsoft Excel Sheet was used for descriptive analysis. Frequency, percentage, mean, and standard deviation were calculated.

## RESULTS

Out of total 144 responses analysed, only 81 (56.3%) participating dental students had known about the term 'impacted teeth' before joining BDS course (Table 1).

**Table 1:** Participants' responses to the questions on impacted teeth.

Questions	Yes, n (%)
Have you visited a dentist before joining BDS programme?	120 (83.3)
If yes, then how often did you visit a dentist?	
• Only once	48 (33.3)
• Yearly/occasionally	44 (30.6)
• Six monthly/regularly	28 (19.4)
• Never	24 (16.7)
Did you know the term 'impacted tooth' before joining BDS course?	81 (56.3)
If yes, how did you come to know about impacted teeth?	
• Did not know	63 (43.8)
• Internet	27 (18.8)
• Dentist	23 (16)
• Friends	14 (9.7)
• Family	12 (8.3)
• Others	5 (3.5)
Is impacted teeth seen in everybody?	5 (3.5)
Among these which impacted teeth you are more familiar with?	
• Third molar	116 (80.6)
• Canine	17 (11.8)
• Incisor	9 (6.3)
• First molar	1 (0.7)
• None	1 (0.7)
Is radiograph necessary for every impacted tooth?	124 (86.1)
Do you know the signs and symptoms of impacted tooth?	101 (70.1)
Is it necessary to remove all impacted teeth?	55 (38.2)
Are you aware of the complications of not removing impacted teeth?	100 (69.4)
Do you know about the types of impacted teeth/impaction?	62 (43.1)

**Figure 1:**
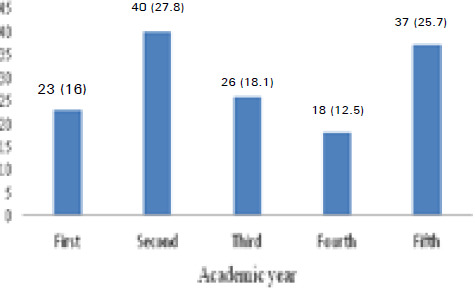
Academic year of participating students, n (%).

The source of information was mostly internet (27, 18.8%) followed by dentist 23 (16%). Among all, 120 (83.3%) had visited a dentist before but only 28 (19.4%) had visited on a regular basis. Most of the participating students were from second year 40 (27.8%) followed by fifth year 37 (25.7%) students (Figure 1).

**Figure 2:**
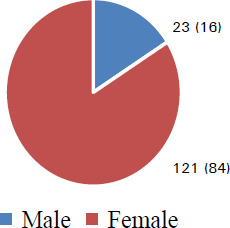
Distribution of participating students according to sex, n (%).

Of them, 116 (80.6%) were familiar with third molar impactions, 62 (43.1%) knew about types of impacted teeth/impaction and 100 (69.4%) were aware of the complications of not removing impacted teeth. There were more female 121 (84%) than male students (Figure 2), with mean age of 21.65±1.539 years (minimum = 19 years; maximum = 25 years; SEM= 0.128).

## DISCUSSION

Impacted teeth are those that fail to erupt in the dental arch within expected time.^3,5^ Lack of adequate space or dental arch length is a common cause for this failure.^6–8^ The impacted teeth may remain embedded in the alveolar bone either completely or incompletely, thus failing to erupt fully into the oral cavity within expected time if its path of eruption is obstructed by bone, soft tissue, or another tooth.^3,5,9^ Impacted teeth can be symptomatic or asymptomatic but when infections of surrounding tissues occur, severe pain result.^6^ A lack of space to allow the teeth to erupt results in a risk of periodontal disease and dental caries, which increases with age. This affects the oral health related quality of life and demands increased attention and awareness by individuals and dental practitioners. Though signs and symptoms of impacted teeth are quite common reasons for visiting a dentist, the authors of this study could not find many studies assessing the knowledge of impacted teeth in regular patients or students.^4^

Impaction of teeth are often incidental clinical or radiographic findings during orthodontic or other oral examinations. Impaction of third molars or other teeth are mostly observed in the 20 to 29 years age group.^10^ Majority of the students were from second year 40 (27.8%) with mean age of 21.65±1.539 years. However, out of 144 participants, only 81 (56.3%) had known about the term 'impacted teeth' before joining BDS course (Table 1). The increased knowledge of impacted teeth, signs and symptoms, management, and any complications can help students prevent and deal with their own problems as well as their peers. Hence, present study was conducted to gauge the level of knowledge regarding impacted tooth among undergraduate dental students of KMCTH, Kathmandu, Nepal.

In current study, 120 (83.3%) participants had visited a dentist before joining the dental course but only 28 (19.4%) students had visited regularly while 24 (16.7%) had never visited any dentist before (Table 1). For the early diagnosis of impacted teeth and prevention of the complications associated with impactions, regular dental check-ups are helpful. Of all impactions, mandibular third molars are most commonly impacted followed by maxillary third molars, maxillary canine, mandibular premolars, mandibular canines, maxillary premolars, maxillary central incisors, and maxillary lateral incisors.^6,8,10^ Most students (116, 80.6%) were familiar with the third molar impactions. This was similar to previous findings and an expected response as despite wide variations among individuals, the third molars remain the most commonly impacted teeth in the human mouth followed by canines.^4,10-12^

Radiographic examinations for the wisdom teeth help to assess the number of impacted teeth, positions, shapes and sizes of the crowns and roots, the surrounding bone and the nerve, which usually runs below the roots of the teeth. Multiple teeth impactions are possible in same individual.^6,8,13^ Studies have shown that though third molars are most commonly impacted tooth, other impactions like premolars and incisors are also observed.^2,5,13,14^ Impaction of the premolars and incisors are relatively rare and when present the cause is often a retained deciduous tooth or the presence of abnormalities like odontoma.^6,15,16^ Associated diseases can be also identified by radiographs, such as cysts and tumours in relation to the teeth.^6,17,18^ About 124 (86.1%) students felt necessary to take radiograph for the impacted tooth for the diagnosis in this study.

It is a good response since, the proximity to adjacent teeth or other vital anatomic structures or pressure on nerves due to impacted teeth can be identified in radiographs and help surgeon with treatment planning and appropriate management of the condition.

The impacted teeth can predispose to periodontal disease and dental caries of adjacent teeth resulting in pain, discomfort, and loss of function.^6^ Hence, ideally all impacted teeth should be removed unless there is some contraindication to the removal.^3,8,9^ In current study, though 101 (70.1%) students knew the sign and symptoms associated with the impacted teeth and 100 (69.4%) BDS students were aware of the complications of not removing impacted teeth, only 55 (38.2%) of the students thought it necessary to remove impacted teeth. Impacted molars are more often than not, source of severe pain from pulpitis due to caries or pericoronitis.^6^ Pressure on the inferior alveolar nerve in very deeply positioned lower third molar impactions may be another reason for pain. Presence of impacted teeth also predispose the adjacent erupted teeth to periodontal disease and caries formation.^6^ Therefore, impacted teeth are often associated with pericoronitis, periodontitis, anomalies like cystic lesions, neoplasm, root resorption and can cause detrimental effects on adjacent tooth and are strongly suggested to be removed.^8,12^ The topmost indication for removal of these teeth impactions includepericoronitis, dental caries, facilitation of orthodontic treatment, periodontal disease, obscure facial pain, root resorption and odontogenic cyst and tumour,and pain of unexplained origin.^3,9^

Impacted mandibular third molar may present with various positions in the bone, the classic positions of the tooth, depending on the direction of the crown or the angulation of the tooth, are: mesioangular, distoangular, vertical, horizontal, buccoangular, linguoangular, and inverted.^6,9,10,19^ Impacted teeth may also be classified according to their depth of impaction, their proximity to the second molar and in relation to the ramus of the mandible. In this study 62 (43.1%) students knew about the different types of impacted teeth which, though not a good number, can be considered satisfactory.

Undergraduate dental students start dealing with patients early in their curricular dental education. This is also the age group commonly suffering from the complications of impacted teeth due to third molar or other teeth incidentally identified during orthodontic and/or radiographic examinations. Problems associated with teeth impactions can interrupt regular duties and activities of students and individuals suffering from it, leading to increase in the rate of absentees for university-based routinesconsequently affecting the learning outcomes. Thus, the increased knowledge regarding impacted teeth, various types of impactions, complications associated, and management can help the BDS students better deal with their own as well as patients' oral health issues. The limitations of the study can be that it was a single centred study with a small sample size.

## CONCLUSIONS

This study shows the knowledge of impacted teeth among undergraduate dental students which is more than previous studies but still the knowledge was observed only in half of the participants. Hence, the undergraduate dental student should be provided with appropriate impacted tooth education supported by practical experience. It is recommended that more detailed information regarding impacted should be included into curriculum for better understanding.
